# Daily life stress is linked to increased glucose levels in individuals with insulin resistance: a real-world assessment

**DOI:** 10.1007/s00125-025-06552-x

**Published:** 2025-10-11

**Authors:** Esther Schrems, Judith R. Gruber, Carmen Schiweck, Alea Ruf, Andreas Reif, Rejane Goldbach, Sharmili Edwin Thanarajah, Silke Matura

**Affiliations:** 1 Department of Psychiatry, Psychosomatic Medicine and Psychotherapy, Goethe University Frankfurt, University Hospital, Frankfurt am Main, Germany; 2 Department of Clinical and Health Psychology, University of Vienna, Vienna, Austria; 3 Fraunhofer Institute for Translational Medicine and Pharmacology ITMP, Frankfurt am Main, Germany; 4 Institute for Biostatistics and Mathematic Modelling, University Medical Centre Frankfurt, Goethe University, Frankfurt am Main, Germany; 5 Max Planck Institute for Metabolism Research, Cologne, Germany

**Keywords:** Ambulatory assessment, Blood glucose, Insulin resistance, Stress

## Abstract

**Aims/hypothesis:**

The bidirectional relationship between stress and diabetes is well documented, with chronic stress increasing the risk of diabetes onset and stress adversely affecting clinical outcomes in individuals with diabetes. However, the impact of daily life stress on glucose levels in insulin-resistant individuals, who are at risk of type 2 diabetes, remains unclear.

**Methods:**

The analysis included 116 participants (62 insulin-resistant [IR] and 54 insulin-sensitive [IS] participants) aged 18–78 years. Insulin resistance was defined by a HOMA-IR index above 2.5. Participants completed three standardised baseline questionnaires to assess their affective state. Using ambulatory assessment, daily life stress and affect were assessed for 3 days while continuous glucose monitoring was conducted for 7 days. Linear mixed-effect models were applied to estimate effects between parameters.

**Results:**

While perceived daily stress was not different between individuals with insulin resistance and control participants, we found a significant positive effect of stress on blood glucose level ($\widehat{\beta }=6.24\times {10}^{-3}$, *p*=0.005) in IR individuals, but not in IS control participants. Additionally, stress levels predicted negative affect in both IR ($\widehat{\beta }=-0.08,$ *p*<0.001) and IS ($\widehat{\beta }=-0.08,$ *p*<0.001) participants.

**Conclusions/interpretation:**

Daily life stress contributes to a significant increase in glucose levels in IR individuals, highlighting the need for tailored interventions to mitigate further deterioration and potential progression to type 2 diabetes. These results underscore the importance of integrating stress management strategies into diabetes prevention in at-risk populations. Ambulatory assessments can serve as monitoring tools for identifying at-risk individuals and for testing the efficacy of targeted interventions.

**Clinical trial registration:**

Registered under https://drks.de/register/de, identifier no. DRKS00022774

**Graphical Abstract:**

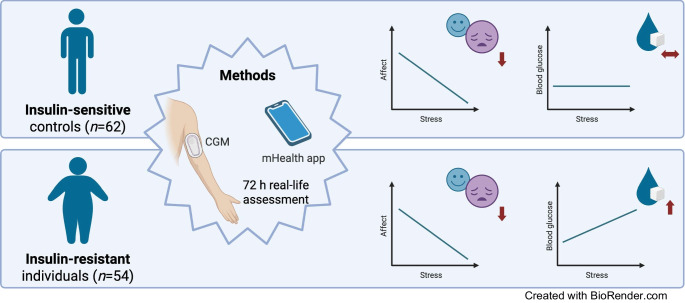

**Supplementary Information:**

The online version of this article (10.1007/s00125-025-06552-x) contains peer-reviewed but unedited supplementary material.


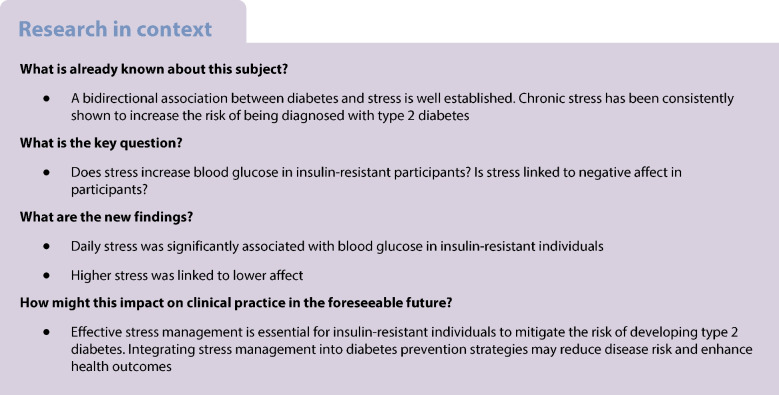


## Introduction

The bidirectional association between diabetes and stress is well established. Chronic stress has been consistently shown to increase the risk of being diagnosed with type 2 diabetes [1]. In turn, individuals with diabetes report higher stress levels, partly due to the disease burden, with a detrimental impact on clinical outcomes [2–4]. Diabetes-related distress is a well-established risk factor for the onset of major depressive disorder (MDD) [3]. The co-occurrence of MDD and diabetes can exacerbate both conditions, resulting in significantly poorer health outcomes [5]. Notably, individuals with type 2 diabetes and comorbid depression face a markedly higher risk of mortality than those with diabetes alone [6]. Therefore, from a clinical perspective, it is crucial to investigate the underlying mechanisms of the interplay between stress, affect and diabetes, to develop effective monitoring strategies and to customise targeted interventions, especially for individuals at risk of developing type 2 diabetes.

Immediate responses to external and internal threats are crucial for survival. In response to acute stress, numerous physiological processes in the body are orchestrated to increase energy availability and optimise mental and physical fitness, which are immediately resolved afterwards. Glucose – the primary energy source – is rapidly released into the bloodstream through the activation of both the sympathetic nervous system and the hypothalamic–pituitary–adrenal axis by stimulating gluconeogenesis and glycogenolysis in the liver [7]. Once the acute stress subsides, glucose is reabsorbed and either utilised or stored to restore equilibrium. Insulin resistance leads to disruptions in these intricate feedback loops and can result in hyperglycaemia [8, 9]. Consequently, daily life stress may have more adverse effects on insulin-resistant (IR) individuals, potentially leading to hyperglycaemia and thereby accelerating the progression of the disease.

While the majority of studies have shown an increase in peripheral glucose levels following exposure to psychological stress among individuals with type 2 diabetes in controlled laboratory conditions [10, 11], the relevance of stress-induced hyperglycaemia in real-world stress scenarios has been hardly explored. Moreover, it remains unclear how daily life stress is related to peripheral glucose levels in participants that have increased insulin levels and are at risk of developing type 2 diabetes.

To close this gap, we analysed the data of 116 either insulin-sensitive (IS [*n*=62]) or IR (*n*=54, HOMA-IR ≥2.5) participants. All participants completed a combined ambulatory assessment of perceived stress and affect, and continuous glucose monitoring for 3 days in a real-life scenario to assess the interaction between stress, affect and glucose levels in insulin resistance.

## Methods

### Participants and study details

The mPRIME study was conducted at the Department of Psychiatry, Psychosomatic Medicine and Psychotherapy of the University Medical Centre Frankfurt in Germany as part of the PRIME project (https://prime-study.eu/), funded through an EU Horizon 2020 grant. The study was approved by the ethics committee of the faculty of medicine of the Goethe University Frankfurt (ethics approval number 20-767, approved 5 September 2020). All participants in the mPRIME study provided written informed consent.

**Fig. 1:**
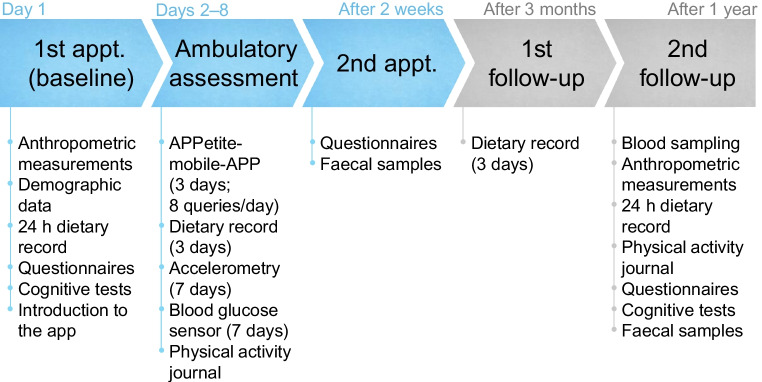
Outline of the study procedure. Appt., appointment

The study design consists of five parts, which are shown in Fig. 1. For the present study, only the first three blocks (in blue in Fig. 1) were considered. These include the first measurement appointment at which the baseline data were taken, a phase of ambulatory assessment and a second in-person appointment.

#### Outline of the study procedure

Potential participants were recruited via advertisements in newspapers, social networks, personal contact with the study staff, doctors’ offices, clinics, pharmacies and gyms. An initial telephone interview for eligibility screening was conducted. Individuals who met the eligibility criteria and were interested in participating were contacted by telephone to arrange an in-person appointment. Participants received detailed feedback on their individual diet, physical activity, cognitive function and blood sugar levels, as well as an incentive payment of €60 and the opportunity to win a €100 voucher.

Between December 2020 and March 2023, a total of 124 people aged between 18 to 78 years who were fluent in German and willing and able to wear monitoring devices (continuous glucose monitoring [CGM] sensor) were included in the study. Gender of participants was determined by self-report during baseline demographic data collection. Both male and female participants were included in the study. Compared with the general adult population of Frankfurt am Main, the sample was broadly similar in age distribution and gender composition. Race and ethnicity data were collected, and the distribution of participants across racial and ethnic groups was representative of the broader adult population of Frankfurt am Main. Socioeconomic indicators (e.g. education, income) were available and suggested that the sample was similar to the general population in educational attainment and income distribution. Individuals were excluded from the study according to the following criteria: any known diagnoses of type 1 diabetes or gestational diabetes; the intake of glucose-lowering medications or insulin; the use of glucocorticoids, weight-reducing medications or special diets; diagnoses of neurological diseases; the diagnosis of lifetime bipolar I disorder, schizophrenia, organic mental disorders or substance abuse; and current pregnancy or breastfeeding. Participants were divided into two groups based on their HOMA-IR index, which was calculated by taking a blood sample during the first appointment screening process in the morning after overnight fast. This index value is determined by the fasting glucose value and the fasting insulin value [12].

The HOMA-IR index is an economical method to ascertain existing insulin resistance [13]. People with a HOMA-IR index ≥2.5 were classified as IR, while those with a HOMA-IR index below 2.5 were considered to be IS [14, 15]. Importantly, existing but untreated type 2 diabetes or prediabetes were not exclusion criteria. For this study, prediabetes was defined according to the American Diabetes Association (ADA) [16] as impaired fasting glucose, specifically a fasting plasma glucose concentration between 5.6 and 6.9 mmol/l, measured from venous blood samples collected in the morning after an overnight fast. After signing the informed consent at the first appointment (baseline), participants were asked to complete questionnaires on demographic data, diet, eating habits, physical activity, impulsivity, depressive symptoms and mental state. In this study, we only included the questionnaires on depressive symptoms and mental state (see the following section for details). In addition, cognitive function tests were carried out, and anthropometric and physiological measurements were taken. These included body height and weight, waist circumference, body fat and water percentage, muscle percentage, bone mass, basic and active metabolic rate, and blood pressure.

The first appointment was followed by an ambulatory assessment for 7 days. During this assessment, participants were provided with a smartphone with a pre-installed application for querying momentary states (the APPetite mobile app) [17], and a CGM (Freestyle Libre Pro IQ sensor, Abbott Diabetes Care, Alameda, CA, USA) was attached to the back of their upper arm. Participants’ physical activity was continuously recorded via a wearable motion tracker (accelerometer) on the non-dominant wrist (Movisens 4 sensor). Participants were asked to report their food intake on 3 consecutive days using the German version of myfood24 [18] and to document their exercise regimen on all 7 days.

Participants were prompted eight times daily over 3 consecutive days, including at least one weekend day (Saturday or Sunday), within a 7 day ambulatory assessment period. Each prompt required them to respond to queries regarding sleep, situational context, affect, stress and food availability. The prompts also included working memory tasks, in which participants had to solve numerical memory-updating tasks (not reported here).

The ambulatory assessment was followed by a second in-person appointment (see Fig. 1) at which the smartphone and the Freestyle Libre Pro IQ sensor were returned. At this point, participants were asked whether the data collected during that period were representative of their usual life.

### Baseline characterisation of participants

Of the 124 participants initially enrolled in the study, data from 116 participants were analysed. The remaining eight participants dropped out due to insufficient time commitment (*n*=6), cognitive impairment (*n*=1) or an unclear diagnosis of diabetes (*n*=1). Of the 116 participants, 62 were included in the IS group (45.2% male, 54.8% female) and 54 individuals were included in the IR group (38.9% male, 61.1% female). A summary of the descriptive statistics is presented in Table 1. From this dataset, nine participants (IS = 5, IR = 4) were excluded from the analysis of the ambulatory data due to missing data points.

**Table 1:** Descriptive statistics, CGM metrics and results of the baseline analysis for depression (BDI-II) and affect (MDBF, good vs bad mood; PANAS-X, positive and negative affect)

Characteristic	IS			IR			
	*n*	Mean	SD	*n*	Mean	SD	*p*
Age (years)	62	45.3	15.4	54	52.2	12.3	0.009
BMI (kg/m^2^)	62	24.7	2.7	54	33.4	8.7	<0.001
HbA_1c_ (mmol/mol)	62	34.9	3.1	54	38.4	5.3	<0.001
HbA_1c_ (%)	62	5.3	0.29	54	5.7	0.49	<0.001
HOMA-IR	62	1.2	0.43	54	4.3	2.3	<0.001
Gender (women/men)	34/28			33/21			0.495
BDI-II	60	4.3	4.9	48	7.0	7.6	0.018
MDBF	61	33.7	5.1	53	33.1	4.9	0.260
PANAS-X positive scale	62	34.7	6.4	53	32.5	6.3	0.034
PANAS-X negative scale	62	16.0	4.8	53	16.2	5.1	0.405
CGM metrics							
TIR 3.9 – 7.8 mmol/l (%)	57	92.8	7.8	53	90.5	10.9	0.221
TAR >7.8 mmol/l (%)	57	6.1	1.3	53	5.5	10.4	0.005
TBR <3.9 mmol/l (%)	57	5.8	7.9	53	3.9	6.0	0.176
CV (%)	57	15.8	3.2	53	17.2	4.3	0.038
MAGE (mmol/l)	57	1.9	0.4	53	2.4	0.9	0.004
Mean SG (mmol/l)	57	0.8	0.2	53	0.9	0.31	0.004

*p* values are from a ${\chi }^{2}$ test for the gender variable and *t* tests otherwise. All CGM metrics reflect the 24 h period; no restriction to daytime or night-time was applied. Due to missing data, the sample size is not equal in all cases

MAGE, mean amplitude of glucose excursions; SG, sensor glucose; TAR, time above range; TBR, time below range; TIR, time in range

### Acquired parameters

Affective state was assessed at baseline and surveyed during the ambulatory assessment period. At baseline, affect was determined with three questionnaires: the Becks Depression Inventory-II (BDI-II) [19], the Positive and Negative Affect Schedule – Expanded Form (PANAS-X) [20, 21] and the Multidimensional Mood Questionnaire (MDBF) [22]. The BDI-II is used to assess the severity of depressive symptoms for the preceding 2 weeks. It is a self-report instrument that consists of 21 items, with each item being rated on a four-point response scale. Using the PANAS-X, participants evaluated the general intensity of 60 different feelings and emotions on a five-point scale ranging from ‘not at all’ to ‘extremely’. The MDBF comprises 24 items, which cover three bipolar dimensions of the current affect (valence [good vs bad mood], alertness vs tiredness and arousal [calmness vs restlessness]). In this study, we only considered the valence dimension (positive vs negative mood). Momentary affect was captured with a short version of the MDBF [23] in the ambulatory assessment. The adapted questionnaire also includes all three mood dimensions, but consists only of eight bipolar items on an eight-point scale, allowing the participants to regularly assess their current affect (eight times per day over 3 days). Eight, one and two participants, respectively, failed to complete the BDI-II, PANAS-X and MDBF questionnaires.

Momentary daily life stress levels were also assessed via an app. Participants were prompted eight times per day over 3 consecutive days to subjectively quantify the stress that they had experienced since the last query on a scale from 0 (not at all) to 100 (very much). Similar to the prompts assessing affect (MDBF), the prompts assessing stress were initiated at eight semi-random time points per day between 08:00 and 22:00. The minimum interval between two prompts was 1 h. Therefore, participants could not predict the exact time of the next prompt and the assessed situation was a better reflection of the participant’s real life. Participants were instructed to respond immediately to the prompt. However, if participants were unable to reply instantly, it was possible to postpone the prompt for 5, 10, 15, 20 or 25 min to avoid missing data and to reduce the participants’ burden.

#### CGM metrics

CGM was conducted using the Freestyle Libre Pro IQ sensor (Abbott Diabetes Care, Alameda, CA, USA). A thin needle permanently protruding from the sensor into the participants’ upper arm records a measurement of the blood glucose level. CGM data were recorded every 15 min for 7 consecutive days. Thus, a total of at least 672 evenly spaced measurements were obtained for each participant. CGM metrics were calculated separately for each valid 24 h period (00:00–23:59) and then averaged across all available days per participant. All computations were performed using the iglu R package without any modifications, applying the default algorithms as described by Broll et al [24]. The following CGM metrics were derived for the baseline analysis: mean sensor glucose (arithmetic mean of all glucose readings per 24 h), CV [(SD/mean glucose) × 100%], time in range (percentage of time with glucose between 3.9 and 7.8 mmol/l), time below range (percentage of time with glucose <3.9 mmol/l), time above range (percentage of time with glucose >7.8 mmol/l) and mean amplitude of glucose excursions (computed following Broll et al [24] using iglu default parameters). All metrics reflect the entire 24 h period; no restriction to daytime or night-time was applied.

### Statistical analyses

Group comparisons for baseline CGM metrics and psychometric parameters (Table 1) were conducted using Welch’s unequal variances *t* test [25]. These descriptive analyses were considered exploratory and hypothesis generating; therefore, no formal correction for multiple testing was applied.

The 3 day measurement period was divided into 2 h intervals and, for each interval where data were available, a statistical average of the measured variables was calculated: for the blood glucose measurements, the mean value was calculated in each 2 h interval, resulting in 12 data points per day. For the measurements of stress and affect, a maximum of eight measurements were carried out (at semi-random time points) throughout each day. If multiple measurements were available in a 2 h interval, the median value was calculated. Only those intervals with data for all three parameters (glucose, stress and affect)—typically five to seven per day—were included in the models. Time spans with missing data were excluded from the analysis. Participants who did not respond to the mobile app queries, who had problems with their CGM measurements or for whom these data were not collected concurrently were excluded entirely from the analysis.

Linear mixed models (LMMs) were conducted to determine the effects of momentary stress on blood glucose and affective state. Non-independence inherent in the data structure was accounted for by correcting the standard error.

To investigate the effect of stress on blood glucose, we fitted the LMM with median stress level as a fixed effect with a fixed intercept. Participant and day were modelled as random effects with random intercepts (1+ participant/day) to account for individual and daily variations. The models were corrected for age and gender. A within-person correlation structure was assumed in the form of an autoregressive process of order 2. For the prediction of blood glucose level, the data on stress were assumed to be autocorrelated. This was confirmed by fitting the LMM under the assumption of no autocorrelation; the modelled residuals show a clear autocorrelation for the fixed factor. Subsequently, the LMM was fitted assuming an autocorrelated structure of order *p*. A variation of *p* provided the best results (lowest Akaike information criterion) for *p*=2. This means that the stress levels are influenced by the levels of the prior two intervals (corresponding to 4 h). Using the same approach, we investigated the impact of stress on affective state. In both cases, we performed the investigation in IR participants; subsequently, we tested the association in IS participants.

All analyses were performed in R statistical software (v4.1.1; [26]). LMMs were calculated via the nlme R package (v3.1-152; [27]). A Bonferroni correction for multiple comparisons was applied at the confirmatory analyses (*n*=4), resulting in an adjusted alpha level of 0.0125.

## Results

In the psychometric characterisation, compared with IS control participants, IR participants reported more depressive symptoms (BDI-II [BDI-II [*t*(76.659) = −2.144, *p*=0.018]) and less positive emotions (positive scale of PANAS-X [*t*(110.961) = 1.842, *p*=0.034]). The valence scale of the MDBF (*p*=0.260) and the negative scale of the PANAS-X (*p*=0.405) did not significantly differ between the two groups (see Table 1).

### Stress was significantly associated with blood glucose only for IR participants

**Fig. 2:**
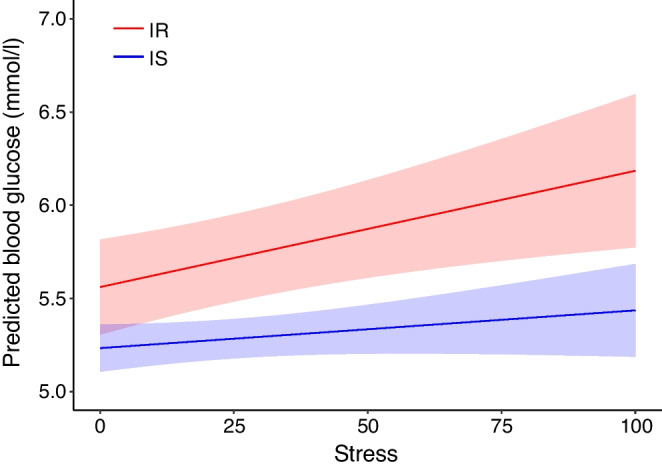
Results of the LMM of the IS group (blue) and the IR group (red). The shaded areas represent the bounds of the 95% CI

Momentary stress was not significantly different between the IS group (mean = 23.96, SD = 13.59) and the IR group (mean = 23.25, SD = 13.67) according to Welch’s *t* test $[t\left(103.03\right)=0.269, p\!\!=\!\!0.789]$. Missing data points in the ambulatory assessment reduced the dataset to ${n}_{\text{IS}}=57$ and ${n}_{\text{IR}}=50$ from the original 116 participants. For the IR participants, momentary stress significantly predicted blood glucose levels ($\widehat{\beta }=6.24\times {10}^{-3}, p\!\!=\!\!0.005$) indicating that higher stress levels were linked to elevated blood glucose in IR participants (Table 2, Fig. 2, ESM Fig. 1). No significant association was found in the IS group ($\widehat{\beta }=2.03\times {10}^{-3}, p\!\!=\!\!0.178$).

**Table 2:** Summary of the LMM relating blood glucose (in mmol/l) to stress

	IS dataset (*n*=57)			IR dataset (*n*=50)		
Predictors	Estimates ($\widehat{\beta }$)	SE	*p*	Estimates ($\widehat{\beta }$)	SE	*p*
Fixed effects						
Intercept	4.63	0.18	<0.001	4.40	0.54	<0.001
Age	1.27 × 10^−2^	3.57 × 10^−3^	<0.001	1.86 × 10^−2^	9.78 × 10^−3^	0.064
Gender (men=1)	3.40 × 10^−2^	0.11	0.754	0.47	0.24	0.062
Stress level	2.03 × 10^−3^	1.50 × 10^−3^	0.178	6.24 × 10^−3^	2.24 × 10^−3^	0.005
Random effects						
${\tau }_{\text{day}}$	0.12			0.18		
${\tau }_{\text{participant}}$	0.37			0.82		

$\tau$ is the estimated SD of the random effects across studies

### Stress was significantly associated with affective state

Momentary stress was a significant predictor of affective state in both the IS and the IR groups (${\widehat{\beta }}_{\text{IS}}={\widehat{\beta }}_{\text{IR}}=-0.08 ,$ *p*<0.001), with higher stress being linked to more negative affect. The results are listed in ESM Table 1.

## Discussion

This study is the first to investigate the relationship between perceived stress and blood glucose levels of IR individuals in a real-life setting using ambulatory assessment including CGM. Although mean momentary stress was comparable between IR and IS individuals, higher levels of stress predicted elevated levels of blood glucose only in IR participants. These findings indicate that daily life stress has an impact on blood glucose dysregulation in individuals with IR, potentially accelerating disease progression. Addressing stress management may therefore be essential to mitigate further deterioration and potential progression to type 2 diabetes.

### Stress is related to an increased risk of diabetes and poorer disease outcomes

It is well established in epidemiological data that persistent stress increases the risk of prediabetes and diabetes [1, 28–32]. In a 13 year prospective study with 5337 participants, Huth et al [1] identified that participants with high job stress at baseline had a 45% increased risk of being diagnosed with type 2 diabetes. Moreover, stress has been demonstrated to increase the risk of complications in individuals with type 2 diabetes, indicating stronger disease progression [33].

### Stress increases blood glucose levels

In this context, the impact of stress on blood glucose homeostasis is potentially the driving force. Goetsch et al [11, 34] had already reported 30 years ago, in a very small sample (*n *= 6–8), increased blood glucose levels after an experimental stress condition in individuals with type 2 diabetes. In line with this, Faulenbach et al [10] found that exposing individuals with type 2 diabetes to moderate psychological stress using the Trier Social Stress Test significantly increased their postprandial blood glucose levels compared with levels without a preceding stressor. Interestingly, this effect was not evident when the participants were exposed to stress in the fasted condition. However, there are also reports from small studies that did not confirm the association between experimental stress and hyperglycaemia in type 2 diabetes [35, 36]. Experimental stress paradigms have the clear advantage that they apply a clearly defined psychological stressor to investigate the physiological response. However, these findings do not easily translate to real-life settings, which is critical when it comes to clinical evaluation and treatment. An early study with only six individuals with type 2 diabetes, who monitored their daily stress and blood glucose through manual assessment, found that mean blood glucose level was increased on days perceived as stressful compared with non-stressful days [11]. Our findings clearly extend these early reports: using ambulatory assessment and CGM, we found that higher perceived stress levels were associated with elevated blood glucose levels in IR participants. This effect was not evident in the IS cohort.

It is crucial to highlight that, unlike the previously mentioned studies on type 2 diabetes, we examined participants with IR (as indicated by the HOMA-IR). Given the elevated type 2 diabetes risk with increasing HOMA-IR [37], our findings have an important clinical value. In these participants, the stress-induced rise in glucose levels might further increase the risk of progression.

### Physiological mechanisms underlying the link between stress and blood glucose levels

The biological mechanisms by which stress leads to increased blood glucose levels in conditions of insulin resistance are not fully understood. Activation of the sympathetic nervous system and of the hypothalamic–pituitary axis and increased levels of glucocorticoids enable rapid energy mobilisation with increased blood glucose availability through gluconeogenesis in the liver and reduced glycogen synthesis in the muscle, which is essential during stress response [38, 39]. Moreover, insulin release is increased while insulin sensitivity is considered to be reduced through reduced GLUT-4-mediated glucose transport in muscle, liver and fat [40, 41]. There are inconsistent findings indicating that both systems are dysregulated in type 2 diabetes, leading to increased glucose availability [8, 9]. This is further exacerbated by the fact that the lack of peripheral insulin action leads to insufficient metabolisation of blood glucose. In addition, persistent stress exposure has been shown to further impact insulin synthesis in the pancreas and insulin action in the periphery [42, 43].

### Stress is associated with negative affect

As expected, we found higher stress levels to be associated with more negative affect in both groups. The impact of stress on emotional well-being may subsequently heighten stress perception and contribute to a detrimental cycle. Moreover, consistent with previous studies, we found that IR participants reported more depressive symptoms than IS control participants [3, 4, 44, 45]. Nevertheless, the level of perceived daily stress was comparable between IR and IS participants.

### Implications of our finding for prevention and treatment

Our research findings illustrate that chronic stress in daily life contributes to an increase in blood glucose levels in IR participants, which might have significant implications on disease progression. This represents a viable target for the implementation of selective preventive and therapeutic strategies. Screening individuals for daily stressors and major life events – and educating them about the link between stress and insulin resistance – therefore offers a promising avenue for targeted prevention and therapy. In addition to the well-recognised lifestyle modifications such as a healthy diet and regular physical exercise that are recommended upon diagnosis of prediabetes and diabetes, techniques aimed at stress reduction may offer a promising strategy for the prevention of hyperglycaemia and the deceleration of disease progression in individuals at risk of type 2 diabetes. Given that prediabetes is a reversible condition [46], targeting stress might be key in mitigating the progression risk and achieving key recovery when combined with the above-mentioned lifestyle adaptions. Mindfulness and acceptance-oriented interventions – including mindfulness-based cognitive therapy (MBCT), mindfulness-based stress reduction (MBSR) and acceptance and commitment therapy (ACT) – are centred on the enhancement of psychological flexibility, awareness and a non-judgemental acceptance of experiences occurring in the present moment, encompassing physical sensations, cognitive thoughts and emotional states. These methodologies assist individuals in managing daily stress by fostering a deeper acceptance of unwanted experiences and by encouraging self-compassion [39]. MBCT, MBSR and ACT are widely recognised psychotherapeutic interventions aimed at addressing diabetes-related distress and they have been demonstrated to have considerable efficacy [47]. Nonetheless, their potential as preventive strategies within at-risk populations has yet to be thoroughly investigated. Hence, we need further investigations on the efficacy of these interventions in preventing type 2 diabetes [39]. In this context, CGM and ambulatory stress assessment can serve as monitoring tools to identify participants at risk and to assess treatment efficacy. We did not conduct gender-stratified analyses due to insufficient subgroup sample sizes within each insulin resistance category, which would have limited statistical power. While this decision was made to avoid underpowered and potentially misleading results, it does mean we cannot determine whether the observed associations differ by gender.

While our findings underscore the importance of stress management in IR individuals, some limitations should be acknowledged. Notably, there was a significant age difference between the IR and IS groups, which reflects the epidemiological reality that insulin resistance increases with age. Although age matching could have reduced between-group variance, it would have compromised the representativeness of our sample. To mitigate this potential confounding factor, all statistical models were adjusted for age. Future studies may benefit from stratified recruitment or subgroup analyses to further isolate age-independent effects. Another limitation is that we did not perform gender-stratified analyses. Although gender was recorded by self-report during baseline assessment, splitting the cohort into subgroups by both insulin resistance status and gender would have resulted in small sample sizes per cell, substantially reducing statistical power and increasing the risk of type II errors. Moreover, the study was not originally powered to detect gender-by-stress or gender-by-glucose interactions, and such exploratory testing would have increased the likelihood of false-positive findings due to multiple comparisons. For these reasons, gender was not included as a stratification variable in the analyses, although it was included as a covariate where appropriate. This choice limits our ability to determine whether the observed stress–glucose associations differ between genders, and thus constrains the generalisability of our results. Future studies with larger, balanced samples should be designed to test potential gender-related effects, given the established biological and psychosocial differences in stress reactivity, insulin resistance and diabetes risk.

## Conclusion

Using ambulatory assessment in everyday life, we were able to show that stress had a significant effect on blood glucose levels in IR individuals. This finding emphasises the need for preventive measures targeting stress levels, especially in IR individuals, to counteract mechanisms that trigger the dysregulation of blood sugar levels and promote disease progression. By integrating stress assessment, CGM insights and stress mitigation techniques into routine care, providers can better guide individuals to avert or delay type 2 diabetes, ultimately improving long-term health outcomes.

## Supplementary Information

Below is the link to the electronic supplementary material.

ESM (PDF 266 KB)

## Data Availability

The data presented in this study are available on Zenodo (10.5281/zenodo.11235373).

## Abbreviations

ACT: Acceptance and commitment therapy
BDI-II: Becks Depression Inventory-II
CGM: Continuous glucose monitoring
IR: Insulin-resistant
IS: Insulin-sensitive
LMM: Linear mixed model
MBCT: Mindfulness-based cognitive therapy
MBSR: Mindfulness-based stress reduction
MDBF: Multidimensional Mood Questionnaire
MDD: Major depressive disorder
PANAS-X: Positive and Negative Affect Schedule – Expanded Form
