# Supplementary material for: Daily life stress is linked to increased glucose levels in individuals with insulin resistance: a real-world assessment
Source: Diabetologia. 2025 Oct 11;68(12):2709–18. doi: 10.1007/s00125-025-06552-x (PMC12594686; doi:10.1007/s00125-025-06552-x)
Supplement: Supplementary file 1 — ESM (PDF 266 KB) [file 125_2025_6552_MOESM1_ESM.pdf]

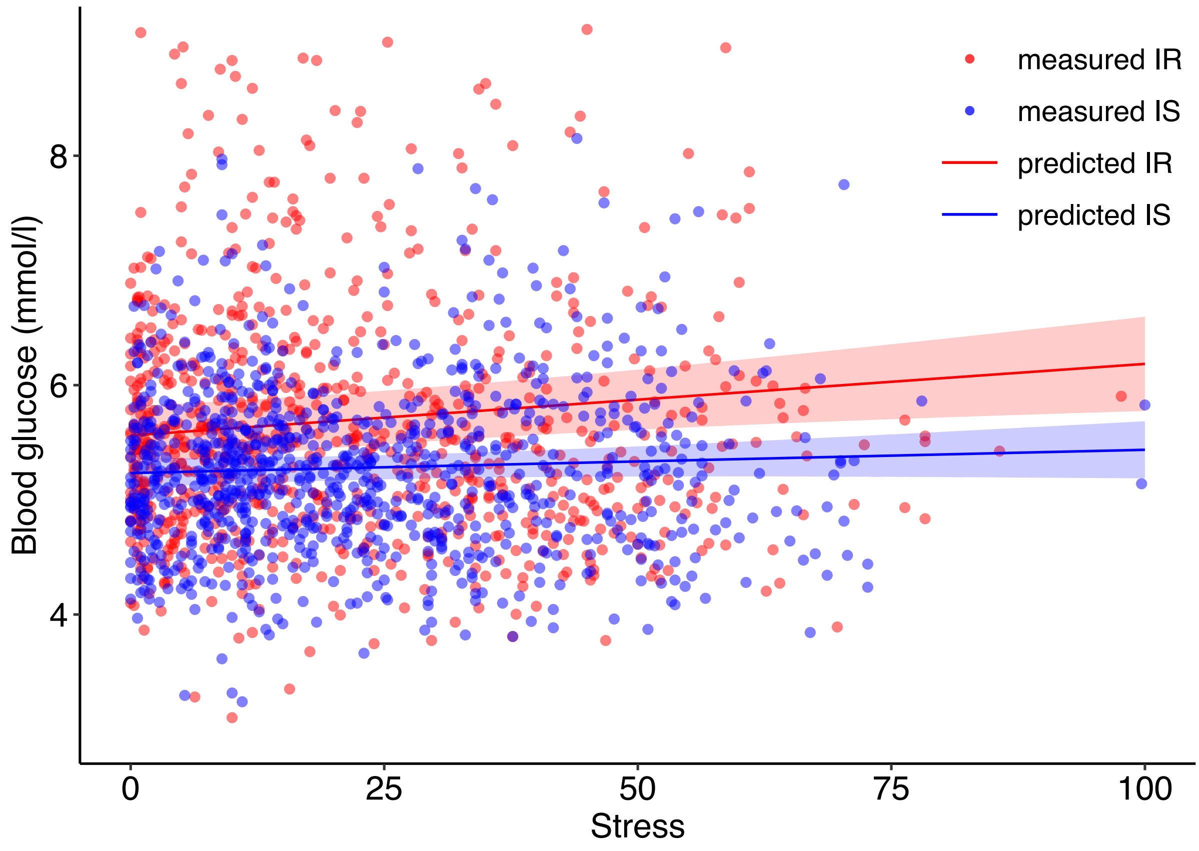

ESM Fig. 1 Results of blood glucose vs. stress for the IS group (blue) and the IR group (red). Circles are the data derived from the ambulatory assessment of blood glucose and stress. Lines are predictions of the LMM. The shaded areas represent the bounds of the 95% CI

ESM Table 1

Summary of the Linear Mixed Models Relating Blood Glucose (in mmol/l) and Stress to Affect.

	**IS dataset** $n = 57$			**IR dataset** $n = 50$		
**Fixed effects**						
Predictors	Estimates ($\hat{\beta}$)	*SE*	*p*	Estimates ($\hat{\beta}$)	*SE*	*p*
(Intercept)	18.58	.85	< .001	19.19	1.01	< .001
Age	1.19×10^{-2}	1.39×10^{-2}	.395	-2.98×10^{-3}	1.67×10^{-2}	.859
Sex (♂ = 1)	6.70×10^{-2}	.42	.874	-.47	.42	.268
Blood glucose (mmol/l)	.13	.11	.205	.10	8.79×10^{-2}	.245
Stress level	-7.83×10^{-2}	5.02×10^{-3}	< .001	-7.98×10^{-2}	5.81×10^{-3}	< .001
**Random effects**						
τ_{day}	.59			.76		
$\tau_{\text{participant}}$	1.44			1.24		

Note. SE = standard error, τ = estimated standard deviation of the random effects across studies, IS = insulin-sensitive, IR = insulin-resistant.
